# The impact of mass-media campaigns on physical activity: a review of reviews through a policy lens

**DOI:** 10.1093/eurpub/ckac085

**Published:** 2022-11-29

**Authors:** Nicolette R den Braver, Enrique Garcia Bengoechea, Sven Messing, Liam Kelly, Linda J Schoonmade, Kevin Volf, Joanna Zukowska, Peter Gelius, Sarah Forberger, Catherine B Woods, J Lakerveld

**Affiliations:** Department of Epidemiology and Data Science, Amsterdam Public Health Research Institutes, Amsterdam University Medical Centres, Amsterdam, The Netherlands; Upstream Team, Amsterdam, The Netherlands; Department of Physical Education and Sport Sciences, Physical Activity for Health Research Cluster, Health Research Institute, University of Limerick, Limerick, Ireland; Research and Innovation Unit, Sport Ireland, Ireland; Department of Sport Science and Sport, Friedrich-Alexander-Universität Erlangen-Nürnberg, Erlangen, Germany; Department of Physical Education and Sport Sciences, Physical Activity for Health Research Cluster, Health Research Institute, University of Limerick, Limerick, Ireland; Medical Library, Vrije Universiteit Amsterdam, Amsterdam, The Netherlands; Department of Physical Education and Sport Sciences, Physical Activity for Health Research Cluster, Health Research Institute, University of Limerick, Limerick, Ireland; Faculty of Civil and Environmental Engineering, Gdansk University of Technology, Gdansk, Poland; Department of Sport Science and Sport, Friedrich-Alexander-Universität Erlangen-Nürnberg, Erlangen, Germany; Leibniz Institute for Prevention Research and Epidemiology—BIPS, Bremen, Germany; Department of Physical Education and Sport Sciences, Physical Activity for Health Research Cluster, Health Research Institute, University of Limerick, Limerick, Ireland; Department of Epidemiology and Data Science, Amsterdam Public Health Research Institutes, Amsterdam University Medical Centres, Amsterdam, The Netherlands; Upstream Team, Amsterdam, The Netherlands

## Abstract

**Background:**

This review of reviews aimed to: (1) summarize the evidence from published reviews on the effectiveness of mass-media campaigns to promote physical activity (PA) or PA-related determinants (intermediate psychological and proximal outcomes) and (2) to identify policy-relevant recommendations related to successful PA campaigns.

**Methods:**

An extensive literature search was performed on 1 March 2021. Reviews that evaluated the impact of campaigns on distal (e.g. PA) and/or proximal outcomes of PA (awareness, knowledge, etc.) and that targeted the general population or subsets were included. Quality of reviews was assessed using the AMSTAR-2 tool. Policy-relevant recommendations were systematically derived and synthesized and formulated as good practice statements. A protocol was registered beforehand (ID: CRD42021249184).

**Results:**

A total of 1915 studies were identified, of which 22 reviews were included. The most consistent evidence was found for the effectiveness of mass-media campaigns on proximal outcomes, while the evidence for distal outcomes was mixed. Good practice statements were derived: (1) to achieve behaviour change, mass-media is an important component of larger, multilevel and multicomponent strategies; (2) mass-media strategies should be coordinated and aligned at local- and national-level and be sustained, monitored and resourced at these levels and (3) media should be tailored to reduce socioeconomic inequalities.

**Conclusions:**

Mass-media can play an important role in the promotion of PA. In general, evidence was more inconsistent for effectiveness on distal outcomes than for proximal outcomes. Policy-relevant recommendations include that mass-media strategies should be resourced, coordinated, aligned, sustained, monitored and evaluated on the local and national level.

## Introduction

Sufficient physical activity (PA) is imperative for public health and the prevention of non-communicable diseases (NCD). However, approximately 25% of adults and 80% of adolescents worldwide do not reach PA guidelines.^1^ The World Health Organization has published ‘best buys’ for tackling NCDs, these include reducing physical inactivity by including community and public education programmes.^2^ Increasing population PA levels is a complex challenge that should incorporate multi-facetted interventions targeting several levels of influence.^2^ It is suggested that such interventions should target individual-level behaviour, alongside environmental level factors, social norms and policy approaches. The International Society for Physical Activity and Health (ISPAH) has formulated eight investments that work for promoting PA.^1^ Mass-media, the core of public education, is one of these investments and aims to raise awareness, transmit consistent and clear messages, and change social norms, to improve population PA^1^

The effect of mass-media campaigns to increase PA has been extensively studied over the past decades and summarized in various reviews.^3–6^ Several outcomes related to campaign effectiveness have been evaluated, including campaign awareness, changes in individual psychological determinants related to PA (e.g. intentions, attitudes and knowledge) as well as changes in PA behaviours.^7^ The available reviews provide various research angles in their summary of the literature, such as a communication perspective, behavioural theory or an evaluation perspective. Thus far, two umbrella reviews have summarized the evidence, where one mainly focused on mass-media on broader health topics (including PA)^8^ and the other focused on PA interventions broader than (but including) mass-media.^9^ Thereby, these do not provide a comprehensive overview of the evidence so far. Moreover, of the many reviews published, few reflect on the potential relevance and implications of their results for policy and policy evaluation. Ineffective campaigns can be the result of inefficient policies, since certain barriers and facilitators can be taken into account in policy recommendations to create impactful campaigns. Therefore, viewing the overall effects at different levels, and analyzing which policy recommendations could improve campaign effects, is pivotal. This information is essential to inform and enable policy makers to weigh their choices based on the best available evidence.^10^ The role of policy is to change systems and create supportive contexts in which programmes and environments collectively can reduce NCD. Policy interventions provide the framework in which programmes, or in this case media campaigns, are tendered, developed, financed or implemented.^11^ Therefore, we aimed (1) to summarize the evidence from published reviews on the effectiveness of mass-media campaigns to promote PA or PA-related determinants (intermediate psychological and proximal outcomes) and (2) to identify policy-relevant recommendations related to successful PA campaigns.

This research is part of the Policy Evaluation Network, a multi-disciplinary research network established for the monitoring, benchmarking and evaluation of policies that affect diet, PA and sedentary behaviour with a standardized approach across Europe (www.jpi-pen.eu).^10^ The policy-relevant recommendations derived from this study will be used to inform the development of the PA environment policy index (PA-EPI), a tool for monitoring and benchmarking government progress in implementing public education policies.^10^

## Methods

### Data sources and searches

This systematic review was conducted in accordance with the Preferred Reporting Items for Systematic Reviews and Meta-Analysis (PRISMA) statement (www.prisma-statement.org) (Online Appendix 5). A comprehensive search was performed on the bibliographic databases PubMed, Embase.com, the Cochrane Library (via Wiley), Cinahl (via Ebsco), SportDiscus (via Ebsco), Scopus and the Web of Science Core Collection, from inception until 1 March 2021. The search was developed in collaboration with a medical librarian (L.S.). Search terms included both controlled terms (MeSH in PubMed and Emtree in Embase) and free text terms. The following terms were used (including synonyms and closely related words) as index terms or free-text words: ‘PA’, ‘policy’, ‘mass media’ and ‘impact’. A search filter was applied to limit results to review studies. The search itself was performed without language restrictions, but only manuscripts were eventually included written in English, Dutch and German (see Inclusion criteria further down). Bibliographies of identified articles were hand-searched for relevant publications. Duplicate articles were excluded. The full search strategies for all databases can be found in Online Appendix 1. The protocol and search strategy used were uploaded to PROSPERO prior to carrying out the study (CRD42021249184^11^).

### Study selection

Two reviewers (N.R.d.B. and J.L.) independently screened titles and abstracts of all identified studies. Subsequently, articles selected for full-text screening were retrieved and assessed for eligibility. Inconsistencies were resolved through consensus. Studies were included if they: (1) Studied mass-media campaigns to promote PA. Mass-media was defined as ‘Messages (either through text or spoken or pictures or videos) distributed via media intended to reach a mass audience. The most common platforms for mass-media are newspapers, magazines, radio, television, billboards, the Internet and social media’^5^; (2) studied change in PA behaviour (direct) or changes in intermediate outcomes including awareness, attitude, beliefs, social norms, intention and knowledge in relation to PA (indirect); (3) presented a summary of evaluation studies, either as systematic review, meta-analysis or narrative review; (4) studied the general population or subsets (such as socio-economic groups and children) and (5) written in English, Dutch or German. Studies were excluded if interventions targeted clinical populations (i.e. specific patient groups).

### Data extraction

Two reviewers (N.R.d.B. and J.L.) performed data extraction according to a standardized protocol. Extracted information included (1) data on review study: aim, in- and exclusion criteria, design, databases, observed results, policy implications and (2) data on primary studies: included campaigns, country, effect, policy level, target population, combined actions and duration. A random subset of extractions (*n* = 5) was cross-checked by the other reviewer (N.R.d.B. and J.L., as appropriate) for quality assurance.

### Risk of bias

Four independent reviewers (N.R.d.B., J.L., S.M. and E.G.B.) performed a risk of bias assessment using the AMSTAR-2 tool for systematic reviews.^13^ Studies were rated as ‘high confidence’ if no more than one non-critical weakness was identified, as ‘moderate’ with >1 non-critical weaknesses, as ‘low’ when one critical weakness was identified (with or without non-critical weaknesses) and as ‘critically low’ when >1 critical weaknesses were identified (with or without non-critical weaknesses).^13^ Details of this tool were described elsewhere.^13^ Since AMSTAR is designed to assess risk of bias in systematic reviews, narrative reviews received a lower score by definition.

### Data synthesis

**Figure 1:**
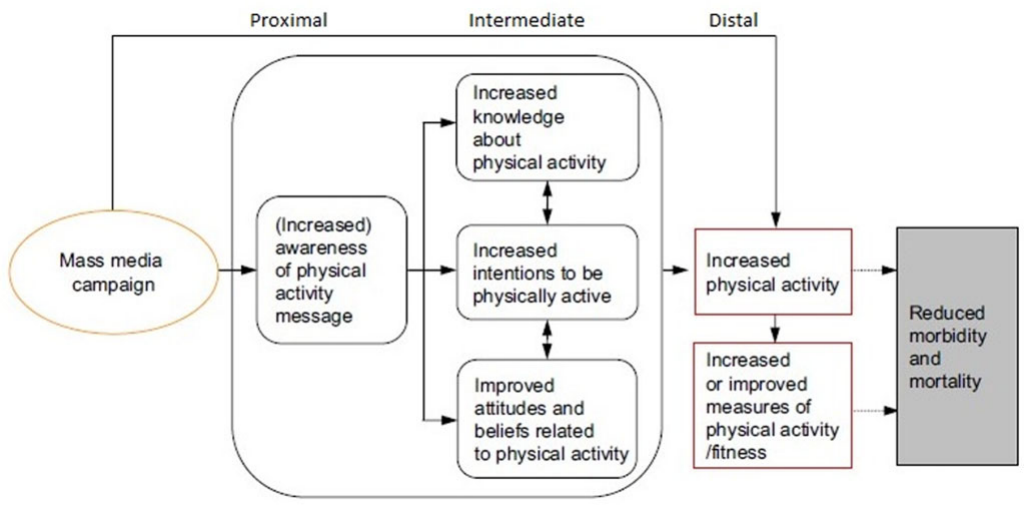
The framework of mass-media campaigns by Brown et al.^7^ with proximal, intermediate and distal outcomes for the individual

We systematically described core characteristics of each review, including the review design, whether the review evaluated PA campaigns only (or a broader range of mass-media campaigns), review aim, overall results, policy implications for evaluation, monitoring and benchmarking and the risk of bias score. We systematically extracted data from each of the included media campaigns and described effectiveness, policy level, country, target population and how often the campaign was identified in the included reviews referred to as overlap in primary studies between reviews. The results were structured according to the levels of outcome based on the analytic framework by Brown et al.^7^ (figure 1). The framework distinguishes the effects of mass-media at various levels: the proximal level (i.e. awareness of messages), intermediate level (i.e. changes in knowledge, intentions and attitudes) and distal outcomes (i.e. changes in PA), which leads to better health outcomes (reduced morbidity and mortality). Also, we classified each campaign according to a typology inspired by Gelius et al., which was initially developed to differentiate policy documents.^14^ We categorized campaigns according to their policy level and their level of impact (see detailed structure in Online Appendix 2). Based on this typology, we differentiated between national and subnational campaigns (policy level) and used the framework by Brown et al. (figure 1) as a guidance for describing the level of impact generated by the study. Overlap of primary studies between reviews was assessed according to methods by Pieper et al.^15^ This was done for overlap of primary studies (publications) as well as at the level of campaigns. The overlap was presented as the corrected covered area (CCA).

## Results

### Search results

**Figure 2:**
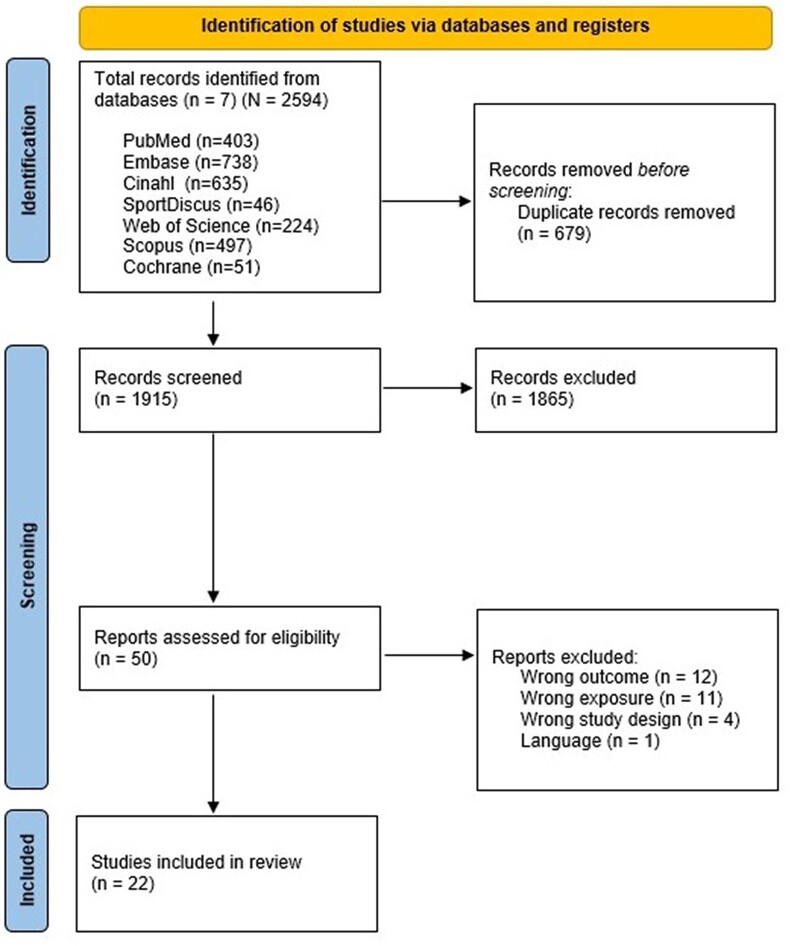
Flowchart of study selection and in- and exclusion

Out of the 1915 identified references, 50 full articles were retrieved and further assessed, with 22 included in our study (figure 2). Fourteen studies received a critically low-quality rating in the risk of bias assessment,^6^^,^^7^^,^^9^^,^^16–26^ three received a low-quality rating,^3^^,^^27^^,^^28^ two received a moderate quality rating^29^^,^^30^ and three received a high-quality rating^8^^,^^31^^,^^32^ (Online Appendix 3). There was a study overlap of 6.8% of campaigns between reviews, with the most cited campaign being Wheeling Walks (covered in 10 reviews) followed by Active Australia (in 8 reviews), Active for life (in 7 reviews) and VERB (in six reviews) (Online Appendix 4).

Table 1 summarizes the characteristics, results, policy implications and risk of bias of the included reviews. Results are discussed below, structured by outcome level.

**Table 1:** Descriptives of review studies, results, policy implications and risk of bias

Author	Year	Review design	Target of included interventions	Review aims	Results	Policy implications	Risk of bias^a^
Abioye^3^	2013	SLR and meta-analysis	PA	To meta-analyze the effect of mass-media campaigns on PA	- Four studies on reducing SB: Pooled RR 1.15 (95% CI: 1.03–1.30), attenuated after exclusion of weak studies - Media campaigns based on social norms were more likely to lead to reduction in SB (RR: 1.33, 95%: 1.01–1.43), risk messaging was less effective (RR: 1.05, 95% CI: 0.92–1.21) - Three studies on targeting sufficient walking were effective: RR = 1.53 (95% CI: 1.25–1.87). - Four studies reported on sufficient PA, no effect (RR = 1.02, 95% CI 0.91–1.14)	NR	Low
Abu-Omar and Rütten^16^	2011	Narrative	PA	To discuss intervention strategies for population level PA promotion and evidence of their effectiveness.	- Inconsistent evidence on effectiveness of mass-media on PA - Moderate evidence of effectiveness mainly in motivated individuals - Campaigns are most suitable to increase knowledge	- Combination with other interventions seems most promising - Address the extent to which campaigns address inequality	Critically low
Anker^17^	2016	Meta-analysis	Health behaviours (4 out of 63 included studies focused on PA)	To meta-analyze the effects of mass-media campaigns on changes in behaviour, knowledge and self-efficacy	- Campaign communities almost uniformly demonstrated more favourable rates of pre- to post-test behaviour change than control communities regardless of the use of theory, implementation of message design factors, channel use or provision of environmental supplements. Larger effects when reasoning for the targeted behaviour was included (CVD, nutrition, PA and safety) - 4 (out of 63) studies on PA - Health campaigns produce a consistent advantage over the absence of campaign messages - Campaigns are predicted to provide a 5% behaviour change	- Recognize complexity of reaching target audience - Communicate what outcome the campaign strives for	Critically low
Brown^7^	2012	SLR	PA	To determine the effect of stand-alone media campaigns to increase PA at population level	- Insufficient evidence for effectiveness of stand-alone media campaigns - Large variations in campaign intensity, duration, dose and reach - Most studies used self-reported measurement of PA (not reporting validity/reliability) - Majority of studies reported on awareness	- Due to lack of effectiveness, campaigns must be used as part of broader multicomponent community-wide interventions to increase awareness, knowledge, and change attitudes and norms - When designed to reach mass public and without evidence, it is unclear to what extent a mass-media campaign reaches most vulnerable groups. Consider audience segmentation - Since awareness is frequently assessed and is a critical component of campaign effectiveness, it is very important to use standard measures to document campaign dose, intensity, duration and reach, as these variables influence message awareness and can affect other distal outcomes	Critically low
Cavill^18^	2004	SLR	PA	To review the effectiveness of mass-media on social norms and PA	- Most immediate outcome of any campaign was increased awareness of campaign message (11/15 reported awareness) - Awareness was usually high (range: 38–97%) - 6/15 campaigns reported intermediate outcomes, three found significant increases - 3/7 studies found increase in intentions when these were targeted - All investigated PA, large variation in measurements, 10 found no significant increases in PA and the others that did were in (presumably highly motivated) subgroups	- Mass-media campaigns have an important role to play as part of a sustained and coordinated multi-level strategy to initially change social norms towards inactivity and then to increase population-level PA - Longer-term strategies are needed, such as those adopted for smoking or HIV, which recognize that social norms do not change overnight, and which invest in change commensurate with the defined needs and time-frames required for change - Funding is often short-time and tends to be defined narrowly	Critically low
Bauman^19^	2009	SLR	PA	To review the effectiveness of mass-media and ‘new media’ campaigns on PA	- Update of study by Cavill, 2004 - New targets identified: adolescents, elderly, low-income groups and non-health-initiated campaigns - Campaign awareness ranges from 31% to 90%, important short-term impact evaluation target - VERB was the most carefully evaluated campaign, which was well branded, had a good process evaluation and showed that high media exposure was associated with improved PA outcomes - Cost-effectiveness studies were lacking	- Sustained government and community support, with sufficient media reach and other programme that support communications campaign contribute to successful campaigns - Collaboration among federal, state and local agencies to develop consistent messages, have a clear and standardized brand and be consistent with national PA guidelines - Integration of mass-media components in long-term PA strategic plans, by state and local public health agencies - Undertake standardized approaches to evaluation to monitor and evaluate campaigns	Critically low
Marcus^6^	1998	SLR	PA	To assess the efficacy of media-based methods on PA	- Recall of messages is high (mean of 70% of participants) - Ability of campaigns to influence PA behaviour remains debatable	- Strategies targeting segments of the population increase efficacy - For the impact of media interventions to be maintained, support systems are required to prompt and support participation over time - Events were more likely to attract participants when held in existing organizations such as workplace - Develop local-level partnerships in implementing large-scale PA campaigns and put resources into local-level services, to support behaviours stimulated by campaigns - Media have crucial role in agenda setting for decision-makers. - Quality control is crucial - Centralized expertise and funding can provide responsibility and resources to implement and evaluate PA programmes.	Critically low
Marshall^20^	2004	SLR	PA	To review the effectiveness of mass-media, print, telephone and website delivered interventions on PA	- Update of review by Marcus, 1998 - Campaigns can result in significant increases of recall/awareness - Limited impact on behaviour	- Focus on regional areas in campaigns, to enable coordinated, collaborative and sustained efforts to be established - Comprehensive approaches resulted in greater behaviour change	Critically low
Finlay^21^	2005	SLR	PA	To review the effectiveness of media interventions on PA	- Update of review by Marcus, 1998 - Positive changes found in recall/awareness - Recall was lower in certain demographic groups - 5/8 studies reported increased PA behaviour, but caution likely selective samples (more motivated) - More sophisticated media approaches to target messages seemed promising (e.g. by assessing stage of change)	- Local-level partnerships and resources required to support behaviour change stimulated by campaign messages - Subgroups need to be targeted in more sophisticated ways	Critically low
Foster^29^	2018	SLR	PA (walking)	To review the effectiveness of population approaches to promote walking and whether there were sustained effects	- Five mass-media with combined approaches (walking groups, pedometers, etc.), two reported long-term effect (12 month) - Natural experiments that combined three approaches—mass-media, community initiatives and environmental change—increased people’s walking	- The challenges of evaluating large-scale population approaches to promote walking reflect the practical and political issues needed to construct a robust research framework for a process where implementation lies outside of scientific control	Moderate
Heath^22^	2009	Narrative	PA	To identify effective or promising public health PA interventions	- Community-wide campaigns represent large-scale, high-intensity, high-visibility programming and often use TV, radio, newspapers and other media to raise programme awareness, disseminate targeted/segmented health messages and reinforce behaviour change - Exemplary interventions include the Stanford Heart Disease Prevention Program and the Wheeling Walks - Mass-media campaigns provide informational approaches and are particularly capable of behaviour change combined with community programming - Mass-media campaigns emerged only recently as promising public health practice - An exemplary mass-media intervention VERB, which targeted ‘tweens’, young people aged 9–13 years, in communities across the USA with mass-media efforts, Internet links and community events and programmes designed to increase and maintain PA - Such interventions are characterized by multiple media, segmented messages and links to community programming	- Use multiple media modes and combine campaigns with other initiatives - Campaigns require tailoring and prioritizing informational, behavioural, social, environmental and policy-level interventions to promote PA, as well as the effective use of social marketing principles - The importance of systematic approaches to intervention programme evaluation needs to be emphasized in public health training programmes - Implementation of routine surveillance of PA and SB by state and local public health agencies, including selected health, environmental and policy correlates, among residents - Allocation of sufficient resources by national, municipal and local public health agencies to effectively inform, educate and empower residents to achieve PA goals - Mobilize community partnerships by national, municipal and local public health agencies to develop effective strategies through informational, social and behavioural and policy and environmental approaches to promoting PA. Provide training and capacity building in partnerships with other community organizations in the use and adaptation of evidence-based interventions - Campaigns contain evidence-informed and focused PA messages tailored to the target audience	Critically low
Heath^9^	2012	Review of reviews	PA	To summarize the effectiveness of PA interventions on health promotion and disease prevention at the national, state and regional and local level	- Mass-media campaigns can lead to change, especially when linked to specific community programmes - One emerging practice is delivery of short informational, instructional and motivational messages about PA at key community sites These are delivered regularly to the target population. This is distinct from mass-media campaigns because the messaging is site-specific and is often delivered by a health educator or communicator	- For some populations, campaigns adapted to the local region and culture work better - Adequately train public health workforce - Capitalize on common points of interest of PA campaigns with transportation, injury reduction, sustainability, energy-use, urban-planning and environmental-protection agendas - Secure sufficient resources - Develop intersectoral partnerships for informational, social and behavioural and policy and environmental approaches to PA promotion - Use evidence-based and promising practice methods for planning and - implementation - Implement and evaluate innovative new interventions to add to the - evidence base - Form partnerships with public health agencies to undertake routine surveillance of PA and SB - Provide training and capacity building in partnership with other community organizations in use and adaptation of evidence-based PA interventions	Critically low
Kahn^32^	2002	SLR	PA	To review the effectiveness of various approaches (informational, behavioural, social, environmental and policy approaches) on PA	- Mass-media campaigns might play important roles in changing awareness of opportunities for and benefits of PA, helping to build support for environmental and policy changes that improve PA behaviour and fitness, or both. This review, however, did not assess the effect of mass-media campaigns on such outcomes	- Use multiple media modes and combine with other initiatives	High
Kite^26^	2018	SLR	Overweight/obesity—includes PA outcomes	To review the evidence on strengths and limitations of overweight and obesity prevention campaigns	- Campaign objectives typically focused on the individual, particularly on increased awareness of the health risks of obesity and obesity-related behaviours - Almost all campaigns included a television component - All campaigns used other media channels, most commonly radio, outdoor or online (including social media and campaign websites), but design and implementation details were limited - Process evaluation results were reported for most campaigns (*n* = 10) but were limited to measures of campaign reach for television - Proximal outcome: All campaigns except one reported on campaign awareness, either campaign recall, recognition or a combination of both - Intermediate outcome: Only two campaigns did not report on intermediate impacts. Five of the remaining ones reported increases in knowledge about the risks of overweight and obesity and SSB consumption - Distal outcome: Behaviours were measured in most campaigns (*n* = 12), with the most common outcomes focusing on PA, SB, dietary behaviours and weight loss. Few campaigns reported any significant behavioural changes. Some campaigns found associations of campaign awareness with behaviours but not behaviour change	- Use multiple media modes and combine with other initiatives - Integrate campaigns with broader prevention strategies that target policy and/or environmental changes - Not only evaluate the campaigns themselves but also the effect of these strategies on individuals, professions, organizations and the community as a whole.	Critically low
Leavy^23^	2011	SLR	PA	To review the effectiveness of mass-media campaigns on proximal, intermediate and distal PA outcomes	- Beyond awareness raising, changes in other outcomes were measured, assessed and reported in varying ways - The review highlighted improvements in evaluation, although limited evidence of campaign effects remain	- Investing in mass-media as part of a comprehensive strategy to increase and sustain regular PA might be promising	Critically low
Yun^27^	2017	SLR	PA	To review the recent literature on mass-media PA campaigns from 2010 to 2016. Specifically, to update the characteristics of the evaluation of campaigns, such as (1) campaign features and promotional activities, (2) inclusion of formative and process evaluation/theoretical framework, (3) evaluation design and sampling and (4) campaigns impacts	- Update of the review by Leavy, 2011 - Results indicated higher awareness pre-post campaign (*n* = 5), as well as compared with control groups (*n* = 3). Three found no effect - Intermediate impact measures were included in 16 articles. Two showed increased pre-post campaign. Four articles showed increased intermediate outcomes in those that were aware of the campaign. Seven articles reported mixed. One article found no significant change - Distal impact was included in 21 articles. Most articles applied self-report questionnaires. Five articles reported significant impact of the campaign on increasing PA behaviour. Six articles reported significant behaviour change based on either proximal or intermediate outcomes (behaviour change only in those that increase awareness or other behavioural determinants). Four articles reported that behavioural change was based on the type of activities or subgroups of the target population. Four articles reported no significant campaign effects on behavioural change - The number of mass-media campaigns continues to grow based on the number of studies published over almost 7 years between January 2010 and September 2016	- More frequent utilization of formative and process evaluation and application of conceptual theory/framework reflect recommendations by previous researchers	Low
Mosdol^31^	2017	SLR	Health behaviours (two out of six included studies focused on PA)	To determine the effects of mass-media interventions targeting adult ethnic minorities with messages about PA, dietary patterns, tobacco use or alcohol consumption to reduce the risk of NCDs	- Six Interventions were included that targeted PA in ethnic minorities. Various adaptations were made, such as language, selected media channels, small media in residential areas, outreach packets through community networks and/or content conveyed by people from target groups. Also, the content was adapted to culture; selected cultural expressions were tested in target group and/or addressed unique cultural barriers - The available evidence is inadequate for understanding whether mass-media interventions targeted towards ethnic minority populations are more effective in changing health behaviours than mass-media interventions intended for the population at large - The studies in this comparison could not distinguish between the impacts of exposure to an intervention, cultural adaptation to an ethnic minority group and choice of mass-media channels to increase reach to the target group	- More research is needed in order to inform practice	High
Pate^28^	2011	SLR	PA	To identify common existing international policies established to increase PA in children and adolescents and to examine the extent to which these policies are supported by scientific evidence	- Studies identified for mass-media were not randomized-controlled trials - Positive results from VERB and Agita supported mass-media/advertising for increasing PA in youth	- Most effective interventions were multi-component - There was strong evidence to support policies focused on Physical Education in school, school environmental policy support and mass-media/advertising	Low
Rütten^24^	2003	Narrative	PA	To examine which types of interventions to promote PA on the population level are effective	- An evaluation of different types of interventions to increase PA yields that mass-media campaigns do not seem to be effective in promoting PA	- Studies that have attempted to modify policies and the environment in - order to increase PA show some promising results	Critically low
Stead^8^	2018	Review of reviews	Health behaviours (seven included studies focused on PA)	To review evidence on the effective use of mass-media in six health topic areas (alcohol, diet, illicit drugs, PA, sexual and reproductive health and tobacco); examine whether effectiveness varies across target populations; identify characteristics of mass-media campaigns associated with effectiveness and identify key research gaps	- There was moderate evidence that mass-media campaigns can reduce SB - Mass-media campaigns were found to increase knowledge and awareness across several topics and to influence intentions regarding PA - Overall, the evidence is mixed but suggests that campaigns can reduce SB	- More rigorous evaluation is warranted of mass-media campaigns, including detailed information on the campaign and exposure - More evidence on cost-effectiveness is needed - The uncontrolled and co-created nature of some new media interventions pose particular evaluation challenges which will require the development of new methodologies - Better understanding of the specific contribution of mass-media campaigns delivered as part of multi-component interventions, including those seeking to influence policy agendas	High
Thomas^25^	2018	SLR	PA	To assess current evidence about the equity impacts of PA mass-media campaigns	PA mass-media campaigns had mostly equal or better impacts for the lowest SES group compared with the highest SES group and less frequently produced worse results for low SES groups	- Tailor PA mass-media campaigns to maximize effectiveness for people from low SES groups and include evaluations that consistently measure equity impacts across a broad range of outcomes - Comprehensive targeted initiatives increase PA, both for socioeconomically disadvantaged and general populations, mass-media campaigns play a role	Critically low
WHO^30^	2009	SLR	PA and diet	Provide a summary of tried and tested diet and PA interventions to support and enable individuals to make healthy choices	- Effective interventions are mass-media campaigns promoting PA with community-based, supportive activities such as programmes in schools and local communities or those associated with policies to address local environmental barriers to participation. Moderately effective interventions are intensive mass-media campaigns using one simple message	- Fill the evidence gaps on effective interventions in low- and middle-income countries - Adapt interventions in low- and middle-income countries to the cultural context and involve community members—both in the formative assessment, intervention design and implementation—for the intervention to work	Moderate

*Note*: SLR, systematic literature review; SB, sedentary behaviour.

a Risk of Bias ratings: ‘high confidence’, ‘moderate’, ‘low’, ‘critically low’.

### Mass-media campaigns and proximal outcomes

Eleven out of 22 reviews reported specifically on awareness and recall as proximal outcomes of PA mass-media campaigns. The earliest review dates from 1998 and reports high campaign recall.^6^ Updates of this review in 2004 and 2005, as well as other reviews, both confirmed that campaigns increased awareness.^7^^,^^8^^,^^18^^,^^20^^,^^21^^,^^23^^,^^26^^,^^27^ Awareness was raised as an important target, since this is the most proximal endpoint and may act as an important step towards effectiveness of campaigns on behavioural outcomes.^32^ Authors of reviews that focused on proximal endpoints argued that effectiveness on more distal outcomes might be harder to detect and more proximal endpoints may therefore be suitable to support policy changes and agenda setting.^32^

### Mass-media campaigns and intermediate outcomes

Seven of 22 reviews reported on outcomes related to changes in knowledge, intentions and attitudes. One review concluded that relatively few primary studies included such intermediate outcomes (6/16),^7^ while a more recent review indicated that such outcomes were more commonly evaluated (16/23).^28^ The reviews showed modest, but usually positive changes in intermediate outcomes, following mass-media campaigns,^7^^,^^8^^,^^18^^,^^23^^,^^26^^,^^28^ see also table 1 and Online Appendix 4.

### Mass-media campaigns and distal outcomes

All included reviews reported on behavioural outcomes. Reviews that were conducted longer ago (1998–2004) highlighted that the lack of studies on PA outcomes was an important research gap and that if it was studied mostly inconsistent or limited impact on PA outcomes was observed.^6^^,^^20^^,^^32^ Six recent reviews (2005–2019) reported positive behavioural outcomes following mass-media campaigns and/or reported promising outcomes when combined with other initiatives.^3^^,^^8^^,^^17^^,^^19^^,^^21^^,^^28^ Campaigns that focused on social norms (rather than risk messaging) were found to be most effective, as were those targeting specific PA behaviours (such as walking).^3^^,17,27^ The campaign most frequently cited was VERB, the successful elements of which were audience segmentation, multiple media use and embedding in existing community programmes (multi-component interventions). Although stand-alone PA media campaigns were found to have insufficient evidence of effectiveness in one review,^7^ another review concluded in more general terms that conducting any kind of campaign was more beneficial than not conducting one, regardless of the theory, methods or channels used.^17^ However, a *post hoc* analysis of studies included in the review of stand-alone mass media campaigns^7^ also found that the more successful campaigns included formative research, audience segmentation, message design, channel placement, process evaluation and theory-based frameworks as part of their campaign design and planning.^33^

About half of the reviews concluded that there was inconsistent evidence with regard to PA improvements following mass-media campaigns. There were some key explanations identified for these inconsistencies. First, some reviews observed that effects were mainly found in motivated (selective) subgroups or in those with significant effects on proximal and intermediate outcomes.^16^^,^^27^^,^^34^ Second, reaching vulnerable subgroups of the population was reported to be a challenge and the extent to which campaigns can be used to address inequalities (e.g. targeting subgroups in tailored ways) should be further explored.^6^^,^^7^^,^^17^ Third, interventions that were combined with other initiatives^16^^,^^19^^,^^23^^,^^24^^,^^29^ seemed to be more promising and effective than stand-alone campaigns.^7^ However, a recent review found only few examples of integrating mass-media into broader prevention strategies.^26^ Fourth, a solid evaluation of population-scale approaches is challenging and there is a lack of consistency in the quality of evaluation methods used across studies of mass media campaigns. Moreover, many studies used self-reported PA measures without commenting on the validity and reliability of these measures.^7^ A solid evaluation and use of monitoring frameworks to evaluate campaigns over a longer term was lacking in many studies, hampering the quality of these studies, potentially preventing them from observing effects.^7^^,^^8^^,^^18^^,^^19^^,^^22^^,^^23^^,^^26^^,^^27^ Longer-term studies that evaluate campaign interventions would also enable investigators to gain a deeper understanding, and better inform policy-makers, about the extent to which changes in PA behaviours resulting from mass-media campaigns are sustainable.

### Policy recommendations

When assessing the reviews through a policy lens, three main topics were identified to inform policy evaluation, monitoring and benchmarking.

First, reviews argued the need for strategies to support long-term and sustained initiatives/strategic plans.^6^^,^^18^^,^^19^^,^^22^ An important mechanism reported to change behaviour with media campaigns is through changing social norms, and because social norms do not change overnight, support for long-term strategies is essential.^18^ A key challenge to sustained and long-term strategies is, however, that funding is often on the short term and allocated too narrowly.^18^ Sustained government and community support, in addition to funding, is a requirement that was also reported in multiple reviews,^18^^,^^19^ as well as integration of mass-media in long-term strategic plans of state and local public health agencies.^19^ State and local agencies should incorporate standardized and routine evaluation and monitoring, according to theoretical frameworks,^6^^,^^9^^,^^19^^,^^22^^,^^23^^,^^27^ which in turn should lead to more solid evidence for the use of mass-media campaigns. In addition to evaluate effectiveness, it would also be valuable to perform economic analysis of costs and benefits. As cost-effectiveness data are often lacking, incorporating it in sustained and systematic monitoring and evaluation schemes would be beneficial.^8^ Finally, evaluation frameworks are generally short-term and narrow (focused on individual behaviours) and therefore often too limited to assess broader impacts that media campaigns may have beyond the individual, also as part of larger combined community interventions.

The second cluster of policy recommendations that was observed across systematic reviews was that mass-media outings should be incorporated in larger combined community interventions and broader prevention strategies to reach the highest impact.^6^^,^^7^^,^^16^^,^^18–20^^,^^22^^,^^26^^,^^28^^,^^32^ Mass-media campaigns help to change awareness, knowledge, attitudes and social norms, laying the foundation for subsequent behaviour change through combined interventions.^7^^,^^18^ Partnerships across multiple levels and organizations/agencies can work well to implement PA campaigns in services and events that stimulate the desired PA behaviours and to enable coordinated, collaborative and sustained efforts.^6^^,^^9^^,^^20^^,^^21^ Also, such collaborations are warranted to create coherent and consistent messaging.^9^^,^^19^ Links to existing organizations could work well locally.^6^ Moreover, local partnerships can help to target messages better to local culture and region.^9^ However, despite the recognized need for mass-media to form part of a broader approach, recent reviews indicated that integration into broader strategies was rare and that policy and/or environmental dimensions were not often integrated with media campaigns.^7,^^8^^,^^26^

The third policy implication concerned audience segmentation. Mass-media messages are inherently designed to reach a mass audience. However, reviews criticize that this is not the most effective approach and that messaging should be tailored, by applying audience segmentation.^6^^,^^7^^,^^9^^,^^16^^,^^17^^,^^21^^,^^31^ Multiple reviews indicated that media messages should be evidence-based.^22^ But the evidence for ethnic minority subgroups is still thin. A few reviews aimed specifically to assess the equity impact of mass-media interventions.^25^^,^^31^ One of these observed that when campaigns focused specifically on low socioeconomic subgroups, they were generally more effective in reaching this group,^31^ while the reach of broadly targeted was equal in high and low socioeconomic groups.^6^^,^^25^ Outcomes of broad campaigns were not large enough to reduce socioeconomic inequalities. More appealing and targeted approaches are needed to maximize effectiveness in strata of the population and evaluating those effects well. As the evidence so far for effectiveness of tailored interventions is thin, this warrants further investigation and evaluation.^25^^,^^31^

## Discussion

This review of reviews compiled the evidence on effectiveness of mass-media campaigns to promote PA or PA-related determinants (intermediate psychological and proximal outcomes) and derived relevant recommendations for policy. The current evidence acknowledges that there are various levels of impact of mass-media campaigns and that campaigns can have a pivotal role in changing social norms and awareness. In general, the more distal the outcomes under study, the smaller and more inconsistent the evidence base for effectiveness. As a result, the current evidence for mass-media campaigns indicates that the effectiveness on PA behaviour is limited. Some policy-relevant characteristics related to successful PA campaigns were identified: (1) to achieve behaviour change, mass-media campaigns are important as a component of larger, multilevel and multicomponent interventions/strategies; (2) mass-media strategies should be coordinated and aligned on the local and national level and should be sustained, monitored, evaluated and resourced at these levels and (3) media should be targeted and tailored to specific population sub-groups in order to better address socioeconomic inequalities.

The evidence observed in the current review of reviews was in line with two earlier umbrella reviews.^8^^,^^9^ Stead *et al.* also indicated impact of campaigns on knowledge and awareness, but mixed results on behavioural effectiveness (smoking, sexual health and PA). Heath *et al.* acknowledged the effectiveness of mass-media campaigns to change behaviour mainly when linked to specific community campaigns.^9^ These conclusions also align with proposed theories on the working mechanisms of mass-media campaigns. Wakefield *et al.* suggest that campaigns can affect PA behaviour either directly or indirectly, for example through agenda setting, social norms and public policy.^35^ Also, the framework by Brown *et al*. focuses narrowly on PA behaviour and assumes that a linear impact of awareness on increased knowledge, intentions, attitudes, beliefs, behaviour change and better health outcomes are needed, if mass-media campaigns are to be effective. However, not all the links in the pathway are that well established. For example, the association between health behaviours and better health outcomes is well established; however, this applies less to the association between behavioural determinants (awareness, knowledge, etc.) and health behaviours. The well-known intention–behaviour gap^36^ indicates that there may be many barriers or facilitators to the translation of such determinants into behaviour. Socio-ecological models suggest that effective policies and supportive environments are among those facilitators.^37^ As indicated in the ‘people and places framework’^38^ and the Global Action Plan on PA (GAPPA),^39^ interventions should not solely focus on individual behaviour change (downstream), but should be part of a larger strategy where physical, socio-cultural, political and economic systems (upstream) work together. Effective policies are thus imperative to the successful use of mass-media campaigns.

From the policy lens that this review took, several good practice statements for mass-media policies were derived. A challenge is that campaign implementation is dependent on political or health agency decision-making^29^; therefore, a tool like the PA-EPI can be important to hold political decision-making accountable. The first and second good practice statements for inclusion in this tool illustrate that coordination of national/subnational strategies as well as public private partnerships, and a focus on long-term monitoring and resourcing at these levels, will enhance evaluation, monitoring and benchmarking of mass-media campaigns. Monitoring will be important to evaluate and recognize successes and failures of mass-media strategies and to assess (a) the reach among specific subgroups, (b) the impact of mass-media campaigns on the long-term and various outcomes and (c) the health impacts of sustained and coordinated initiatives. This requires policies to be developed with a longer-term focus and monitoring and surveillance systems that capture indicators of effectiveness and reach. Also, monitoring and sustained efforts will play a crucial role in determining cost-effectiveness of mass-media campaigns and combined efforts and play a role in supporting long-term strategy plans^8^^,^^16^^,^^19^. Cost-effectiveness studies were outside the scope of the present review, but one previous umbrella review assessed cost-effectiveness of PA interventions (including mass-media).^40^ This review supported the cost-effectiveness of mass-media, but also acknowledged the debate of overall effectiveness to change behaviour through mass-media alone. However, the WHO has identified public education and awareness campaigns (incl. mass-media campaigns) combined with other community-based education, motivational and environmental programmes, to be a ‘best buy’ for reducing physical inactivity.^2^ This supports the need for collaboration at the local and national level as well as public private partnerships, and sustained actions to make these initiatives impactful, as outlined in our second good practice statement. It should also be noted that we did not perform exclusions of reviews based on funding of included interventions, while privately funded interventions may have competing interests as opposed to public funded interventions. We did choose to include reviews that selected both types of interventions, to be able to include a perspective on partnerships on multiple levels. We conclude that public–private partnerships can work well when collaboration and interests of programmes are well aligned. Nevertheless, we included the funding sources per intervention in Online Appendix 4, to be transparent on funding sources of each included intervention. Also, focus on upstream determinants of PA and outcomes of mass-media campaigns, and incorporating this in monitoring frameworks, will provide a more holistic view of the effects reached by combined efforts and mass-media, as highlighted by good practice statement 1. Thirdly, tailoring of mass-media to specific population sub-groups emerged as good practice in the present review. Groups with a low socio-economic status are difficult to reach and these are the groups with highest potential gains to be made in terms of health impact. In many of the included reviews, this tailoring was raised as an important research gap and the existing evidence base is thin. While mass-media campaigns in themselves will likely not bridge and eliminate the socio-economic disparities in health, tailoring existing initiatives can increase access to health-enhancing activities and initiatives for lower socio-economic groups.

This review of reviews should be interpreted in light of some limitations and strengths. First, it included only published reviews but not grey literature and by design, the most recent primary studies have not been included. Also, the quality of the evidence in a review depends on the quality of the included studies. We saw that, in general, older reviews scored lower in terms of quality, which is not surprising given that standardization of literature searching, appraisal and reporting has only become common practice over the last decade. Moreover, review studies did not always adequately specify whether proximal determinants referred to the awareness of the campaign or the health issue (i.e. determinant(s) of PA behaviour or PA behaviour); therefore, we could not provide these details in the present review. One additional limitation is that current reviews often recommend that mass-media campaigns should be included as one component of larger, multilevel and multicomponent interventions/strategies. However, policy-makers may want to know what additional components should be added to a mass media multicomponent campaign and/or what is the impact each individual component may contribute to behaviour change. The research literature does not provide direct answers to this question. It may make good common sense, though, for policy-makers to engage community members or the intended audience of the mass-media campaign, to help tailor the additional components of the multicomponent intervention, based on their needs and preferences. This may be especially important when planning and implementing interventions to address health and economic disparities.

Despite these limitations, the review provides a comprehensive overview of the state of the art in the field of mass-media for PA promotion, as well as important and concrete entry points for policy regarding the enhancement of PA through mass-media. The evidence showed that mass-media can play an important role in the promotion of PA, as it can clearly impact on important determinants of PA and might contribute to enhancing PA behaviours—although the latter may only be expected when media outings take place alongside additional PA promotion approaches.

## Supplementary data

Supplementary data are available at *EURPUB* online.

## Funding

The PEN project is funded by the Joint Programming Initiative (JPI) ‘A Healthy Diet for a Healthy Life’, a research and innovation initiative of EU member states and associated countries. The funding agencies supporting this work are (in alphabetical order of participating countries): Germany: Federal Ministry of Education and Research (BMBF); Ireland: Health Research Board (HRB); The Netherlands: The Netherlands Organisation for Health Research and Development (ZonMw); New Zealand: The University of Auckland, School of Population Health and Poland: The National Centre for Research and Development (NCBR).

## Conflicts of interest

The authors declare that they have no known competing financial interests or personal relationships that could have appeared to influence the work reported in this paper.

Key points

- The current review of reviews confirmed the various levels of impact of mass-media campaigns on PA and that campaigns can have a pivotal role in changing social norms and awareness.
- To achieve behaviour change, mass-media campaigns are important components of larger, multilevel and multicomponent interventions/strategies.
- Mass-media strategies should be coordinated and aligned on the local and national level and should be sustained, monitored, evaluated and resourced at these levels.
- Mass-media should be targeted and tailored to specific population sub-groups in order to better address socioeconomic inequalities.

## Supplementary Material

ckac085_Supplementary_Data
