# Supplementary material for: The impact of mass-media campaigns on physical activity: a review of reviews through a policy lens
Source: Eur J Public Health. 2022 Nov 29;32(Suppl 4):iv71–83. doi: 10.1093/eurpub/ckac085 (PMC9706123; doi:10.1093/eurpub/ckac085)
Supplement: ckac085_Supplementary_Data [file ckac085_supplementary_data.docx]

# Supplementary material

## Appendix 1: Search strategy

## PubMed Search History March 1, 2021

| **Search** | **PubMed Query – March 1, 2021** | **Results** |
| --- | --- | --- |
| #7 | #5 AND #6 | 403 |
| #6 | (("Review Literature as Topic"[Mesh] OR "Review"[Publication Type] OR "Meta-Analysis as Topic"[Mesh] OR “review*”[tiab] OR “meta-analys*”[tiab] OR "Meta-Analysis "[Publication Type] OR systematic[sb]) NOT ("Letter"[Publication Type] OR "Editorial"[Publication Type] OR "Comment"[Publication Type])) | 3,821,331 |
| #5 | #1 AND #2 AND #3 AND #4 | 1,274 |
| #4 | "Evaluation Study" [Publication Type] OR "Evaluation Studies as Topic"[Mesh] OR "Program Evaluation"[Mesh] OR "evaluat*"[tiab] OR "impact"[tiab] OR "appraisal"[tiab] OR "effect*"[tiab] OR "assessment*"[tiab] | 11,133,978 |
| #3 | "Mass Media"[Mesh] OR "Communications Media"[Mesh] OR "Social Media"[Mesh] OR "public education"[tiab] OR "mass communication"[tiab] OR "social marketing"[tiab] OR broadcast*[tiab] OR "media"[tiab] | 608,636 |
| #2 | "Policy"[Mesh:NoExp] OR "Fiscal Policy"[Mesh] OR "Social Control Policies"[Mesh] OR "Policy Making"[Mesh] OR "policy"[tiab] OR "policies"[tiab] OR "national framework*"[tiab] OR "legislation"[tiab] OR campaign*[tiab] OR (strateg*[tiab] AND (prevent*[tiab] OR policy[tiab] OR policies[tiab])) | 622,197 |
| #1 | "Exercise"[Mesh] OR "Sedentary Behavior"[Mesh] OR "physical activit*"[tiab] OR "physical inactivit*"[tiab] OR "physical education"[tiab] OR "sedentar*"[tiab] OR "sitting"[tiab] OR "healthy lifestyle"[tiab] OR "healthy life-style"[tiab] OR "health initiative*"[tiab] OR "exercis*"[tiab] OR "Sports"[Mesh] OR sport*[tiab] OR walk[tiab] OR walking[tiab] OR cycle[tiab] OR cycling[tiab] OR cyclist*[tiab] OR cycles[tiab] OR bicycle*[tiab] OR bike[tiab] OR bikes[tiab] OR biking[tiab] OR move[ti] OR "play"[ti] | 1,330,100 |

## Embase.com Search History March 1, 2021 (738)

| **Search** | **Embase.com Query – March 1, 2021** | **Results** |
| --- | --- | --- |
| #8 | #6 AND #7 | 738 |
| #7 | (('systematic review'/exp OR 'meta analysis'/exp OR ‘review*’:ab,ti,kw OR ‘meta-analys*’:ab,ti,kw) NOT  ('conference abstract'/it OR 'conference review'/it OR 'editorial'/it OR 'erratum'/it OR 'letter'/it OR 'note'/it OR 'short survey'/it)) | 2,462,352 |
| #6 | #5 NOT ('conference abstract'/it OR 'conference review'/it) | 3,176 |
| #5 | #1 AND #2 AND #3 AND #4 | 4,125 |
| #4 | 'evaluation study'/exp OR 'program evaluation'/exp OR evaluat*:ti,ab,kw OR effect*:ti,ab,kw OR appraisal:ti,ab,kw OR impact:ti,ab,kw OR assessment*:ti,ab,kw | 13,856,960 |
| #3 | 'public education'/exp OR 'mass medium'/exp OR 'mass communication'/exp OR 'social marketing'/exp OR 'public education':ti,ab,kw OR 'mass communication':ti,ab,kw OR 'social marketing':ti,ab,kw OR broadcast*:ti,ab,kw OR 'media':ti,ab,kw | 1,092,511 |
| #2 | 'policy'/de OR 'public policy'/exp OR 'health program'/exp OR 'public health campaign'/exp OR ‘policy':ti,ab,kw OR ‘policies’:ti,ab,kw OR ‘national framework':ti,ab,kw OR ‘legislation’:ti,ab,kw OR ‘campaign’:ti,ab,kw OR ((strateg* AND (prevent* OR policy OR policies)):ti,ab,kw) | 898,944 |
| #1 | 'physical activity'/exp OR 'physical activity' OR 'sedentary lifestyle'/exp OR 'sedentary lifestyle' OR 'physical education'/exp OR 'physical education' OR 'healthy lifestyle'/exp OR 'healthy lifestyle' OR 'sport'/exp OR 'sport':ti,ab,kw OR 'exercise'/exp OR 'exercise' OR 'physical activit*':ti,ab,kw OR 'physical inactivit*':ti,ab,kw OR 'physical education':ti,ab,kw OR sedentar*:ti,ab,kw OR sitting:ti,ab,kw OR 'healthy lifestyle':ti,ab,kw OR 'healthy life style':ti,ab,kw OR 'health initiative':ti,ab,kw OR exercis*:ti,ab,kw OR sport*:ti,ab,kw OR walk:ti,ab,kw OR walking:ti,ab,kw OR cycle:ti,ab,kw OR cycling:ti,ab,kw OR cyclist*:ti,ab,kw OR cycles:ti,ab,kw OR bicycle*:ti,ab,kw OR bike:ti,ab,kw OR bikes:ti,ab,kw OR biking:ti,ab,kw OR move:ti OR play:ti | 2,034,764 |

## Scopus Search History March 1, 2021 ,

| **Search** | **Scopus Query – March 1, 2021** | **Results** |
| --- | --- | --- |
| #7 | #5 AND #6 | 497 |
| #6 | TITLE-ABS-KEY (“review*” OR “meta-analys*”) | 5,801,760 |
| #5 | #1 AND #2 AND #3 AND #4 | 2,097 |
| #4 | TITLE-ABS-KEY (evaluat* OR {effect} OR {appraisal} OR {impact} OR assessment*) | 20,320,350 |
| #3 | TITLE-ABS-KEY ({media} OR “public education” OR “mass communication” OR “social marketing” OR broadcast*) | 2,240,526 |
| #2 | TITLE-ABS-KEY ({policy} OR {policies} OR “national framework” OR {legislation} OR campaign*) OR TITLE-ABS-KEY (strateg* AND (prevent* OR policy OR policies OR campaign*)) | 2,218,146 |
| #1 | TITLE-ABS-KEY ("physical activit*" OR "physical inactivit*" OR "physical education” OR sedentar* OR {sitting} OR “healthy lifestyle” OR “healthy life-style” OR “health initiative” OR excercis* OR sport* OR [1] OR {walking} OR {cycle} OR {cycling} OR cyclist* OR {bicycle} OR {bike} OR {bikes} OR {biking}) OR TITLE (play OR move) | 2,566,315 |

## Cinahl (Ebsco) Search History March 1, 2021

| **Search** | **Cinahl (Ebsco) Query – March 1, 2021** | **Results** |
| --- | --- | --- |
| S7 | S5 AND S6 | 561 |
| S6 | MH ("Systematic Review" OR "Meta Analysis") OR TI (“review*” OR “meta-analys*”) OR AB (“review*” OR “meta-analys*”) |  |
| S5 | S1 AND S2 AND S3 AND S4 | 1,413 |
| S4 | MH ("Evaluation Research+" OR "Program Evaluation") OR TI (evaluat* OR effect* OR appraisal OR impact OR assessment*) OR AB (evaluat* OR effect* OR appraisal OR impact OR assessment*) | 635 |
| S3 | MH ("Communications Media+" OR "Social Media+") OR TI (“public education” OR “mass communication” OR “social marketing” OR media OR broadcast*) OR AB (“public education” OR “mass communication” OR “social marketing” OR media OR broadcast*) | 642,999 |
| S2 | MH ("Public Policy+" OR "Health Policy Studies" OR "Health Policy+" OR "Policy Making") OR TI (policy OR policies OR “national framework” OR legislation OR campaign* OR (strateg* AND (prevent* OR policy OR policies OR campaign*))) OR AB (policy OR policies OR “national framework” OR legislation OR campaign* OR (strateg* AND (prevent* OR policy OR policies OR campaign*))) | 293,379 |
| S1 | MH ("Exercise+" OR "Life Style, Sedentary+" OR "Physical Education and Training+" OR "Sports+" OR "Life Style Changes") OR TI (exercis* OR “physical activit*” OR “physical inactivit*” OR sedentar* OR “physical education” OR play OR sitting OR “healthy lifestyle” OR “healthy life-style” OR “health initiative*” OR sport* OR walk OR walking OR cycle OR cycling OR cyclist* OR cycles OR bicycle* OR bike OR bikes OR biking OR move) OR AB ( exercis* OR “physical activit*” OR “physical inactivit*” OR sedentar* OR “physical education” OR sitting OR “healthy lifestyle” OR “healthy life-style” OR “health initiative*” OR sport* OR walk OR walking OR cycle OR cycling OR cyclist* OR cycles OR bicycle* OR bike OR bikes OR biking) | 678,282 |

## SportDiscus (Ebsco) Search History March 1, 2021

| **Search** | **SportDiscus (Ebsco) Query – March 1, 2021** | **Results** |
| --- | --- | --- |
| S7 | S5 AND S6 | 46 |
| S6 | TI (“review*” OR “meta-analys*”) OR AB (“review*” OR “meta-analys*”) OR KW (“review*” OR “meta-analys*”) |  |
| S5 | S1 AND S2 AND S3 AND S4 | 329 |
| S4 | TI (evaluat* OR effect* OR appraisal OR impact OR assessment*) OR AB (evaluat* OR effect* OR appraisal OR impact OR assessment*) OR KW (evaluat* OR effect* OR appraisal OR impact OR assessment*) | 129,648 |
| S3 | DE ("MASS media") OR TI (“public education” OR “mass communication” OR “social marketing” OR broadcast* OR media) OR AB (“public education” OR “mass communication” OR “social marketing” OR broadcast* OR media) OR KW (“public education” OR “mass communication” OR “social marketing” OR broadcast* OR media) | 31,791 |
| S2 | DE ("HEALTH promotion" OR "HEALTH education") OR TI (policy OR policies OR “national framework” OR legislation OR campaign* OR (strateg* AND (prevent* OR policy OR policies OR campaign*))) OR AB (policy OR policies OR “national framework” OR legislation OR campaign* OR (strateg* AND (prevent* OR policy OR policies OR campaign*))) OR KW (policy OR policies OR “national framework” OR legislation OR campaign* OR (strateg* AND (prevent* OR policy OR policies OR campaign*))) | 66,790 |
| S1 | DE ("PHYSICAL activity" OR "PHYSICAL education" OR "SEDENTARY lifestyles" OR “SPORTS” OR “EXERCISE”) OR TI ("physical activit*" OR “physical inactivit*” OR play OR sedentar* OR sitting OR “healthy lifestyle” OR “healthy life style” OR “health initiative” OR exercis* OR fitness OR sport* OR walk OR walking OR cycle OR cycling OR cyclist* OR cycles OR bicycle* OR bike OR bikes OR biking OR move) OR AB ("physical activit*" OR “physical inactivit*” OR sedentar* OR sitting OR “healthy lifestyle” OR “healthy life style” OR “health initiative” OR exercis* OR fitness OR sport* OR walk OR walking OR cycle OR cycling OR cyclist* OR cycles OR bicycle* OR bike OR bikes OR biking) OR KW ("physical activit*" OR “physical inactivit*” OR sedentar* OR sitting OR “healthy lifestyle” OR “healthy life style” OR “health initiative” OR exercis* OR fitness OR sport* OR walk OR walking OR cycle OR cycling OR cyclist* OR cycles OR bicycle* OR bike OR bikes OR biking*) | 332 |

## Web of Science Core Collection Search History March 1, 2021

| **Search** | **Web of Science Core Collection Query – March 1, 2021** | **Results** |
| --- | --- | --- |
| #7 | #5 AND #6 | 224 |
| #6 | TS=(“review*” OR “meta-analys*”) | 2,647,577 |
| #5 | #1 AND #2 AND #3 AND #4 | 1,238 |
| #4 | TS = (evaluat* OR effect* OR “appraisal” OR “impact” OR assessment*) | 17,238,503 |
| #3 | TS= (“media” OR “public education” OR “mass communication” OR “social marketing” OR broadcast*) | 738,112 |
| #2 | TS= (“policy” OR “policies” OR “national framework” OR legislation OR campaign* OR (strategy* AND (prevent* OR “policy” OR “policies”))) | 1,127,391 |
| #1 | TS= ("physical activit*" OR "physical inactivit*" OR "physical education" OR sedentar* OR “sitting” OR “healthy lifestyle” OR “healthy life-style” OR “health initiative” OR exercis* OR sport* OR “walk” OR “walking” OR “cycle” OR “cycling” OR cyclist* OR “bicycle” OR “bike” OR “bikes” OR “biking”) OR TI= (play OR move) | 2,036,101 |

## The Cochrane Library (Wiley) Search History March 1, 2021

| **Search** | **The Cochrane Library Query – March 1, 2021** | **Results** |
| --- | --- | --- |
| #6 | Selection: Cochrane Reviews | 51 |
| #5 | #1 AND #2 AND #3 AND #4 | 183 |
| #4 | (evaluat* OR effect* OR appraisal OR impact OR assessment*):ti,ab,kw | 1,243,819 |
| #3 | (media OR “public education” OR “mass communication” OR “social marketing” OR broadcast*):ti,ab,kw | 20637 |
| #2 | (“policy” OR “policies” OR “national framework” OR legislation OR campaign* OR (strategy* AND (prevent* OR “policy” OR “policies”))):ti,ab,kw | 21935 |
| #1 | (physical NEXT activit* OR physical NEXT inactivit* OR physical NEXT education OR sedentar* OR “sitting” OR “healthy lifestyle” OR “healthy life-style” OR “health initiative” OR exercis* OR sport* OR “walk” OR “walking” OR “cycle” OR “cycling” OR cyclist* OR “bicycle” OR “bike” OR “bikes” OR “biking”):ti,ab,kw OR (play OR move):ti | 180497 |

## Appendix 2: AMSTAR-2 Risk of bias scoring sheet per review

(light-grey are critical domains)

| **Amstar Item** |  | **Abioye, 2013** | **Abu-Omar, 2012** | **Anker, 2006** | **Bauman, 2009** | **Brown, 2012** | **Cavill, 2004** | **Finlay, 2005** | **Foster, 2018** | **Heath, 2009** |
| --- | --- | --- | --- | --- | --- | --- | --- | --- | --- | --- |
| 1. Did the research question and inclusion criteria for the review include the components of PICO? |  | no | no | yes | no | yes | no | no | yes | no |
| 2. Did the report of the review contain an explicit statement that the review methods were established prior to the conduct of the review and did the report justify any significant deviations from the protocol? |  | no | no | no | no | yes | no | no | partial yes | no |
| 3. Did the review authors explain their selection of the study designs for inclusion in the review? |  | yes | no | no | no | yes | yes | no | no | no |
| 4. Did the review authors use a comprehensive literature search strategy? |  | yes | no | partial yes | yes | yes | partial yes | partial yes | yes | no |
| 5. Did the review authors perform study selection in duplicate? |  | yes | no | yes | no | yes | no | no | yes | no |
| 6. Did the review authors perform data extraction in duplicate? |  | no | no | no | no | yes | no | no | yes | no |
| 7. Did the review authors provide a list of excluded studies and justify the exclusions? |  | yes | no | no | no | no | no | no | yes | no |
| 8. Did the review authors describe the included studies in adequate detail? |  | yes | no | no | partial yes | partial yes | yes | partial yes | yes | no |
|  |  |  |  |  |  |  |  |  |  |  |
| 9. Did the review authors use a satisfactory technique for assessing the risk of bias (RoB) in individual studies that were included in the review? | **RCT's** | Includes only NRSI | Includes only NRSI | no | no | no | no | Includes only NRSI | Includes only NRSI | no |
|  | **NSRI** | yes | no |  |  |  |  | no | partial yes |  |
| 10. Did the review authors report on the sources of funding for the studies included in the review? |  |  |  |  |  |  |  |  |  |  |
| 11. If meta-analysis was performed did the review authors use appropriate methods for statistical combination of results? | **RCT's** |  |  |  |  | No meta-analysis conducted | No meta-analysis conducted | No meta-analysis conducted | No meta-analysis conducted | No meta-analysis conducted |
|  | **NRSI** | yes | No meta-analysis conducted | yes | No meta-analysis conducted | No meta-analysis conducted | No meta-analysis conducted | No meta-analysis conducted | No meta-analysis conducted | No meta-analysis conducted |
| 12. If meta-analysis was performed, did the review authors assess the potential impact of RoB in individual studies on the results of the meta-analysis or other evidence synthesis? |  | yes | No meta-analysis conducted | no | No meta-analysis conducted | no | No meta-analysis conducted | No meta-analysis conducted | No meta-analysis conducted | No meta-analysis conducted |
| 13. Did the review authors account for RoB in individual studies when interpreting/ discussing the results of the review? |  | yes | NA | NA | NA | NA | NA | NA | yes | NA |
| 14. Did the review authors provide a satisfactory explanation for, and discussion of, any heterogeneity observed in the results of the review? |  | yes | yes | no | no | yes | yes | no | no | no |
| 15. If they performed quantitative synthesis did the review authors carry out an adequate investigation of publication bias (small study bias) and discuss its likely impact on the results of the review? |  | yes | No meta-analysis conducted | yes | No meta-analysis conducted | No meta-analysis conducted | No meta-analysis conducted | No meta-analysis conducted | No meta-analysis conducted | No meta-analysis conducted |
| 16. Did the review authors report any potential sources of conflict of interest, including any funding they received for conducting the review? |  | yes | yes | no | no | yes | no | yes | yes | no |

Appendix 2 (continued)

| **Amstar Item** |  | **Heath, 2012** | **Kahn, 2002** | **Kite, 2018** | **Leavy, 2011** | **Marcus, 1998** | **Marshall, 2004** | **Yun, 2017** | **Mosdol, 2017** | **Pate, 2011** |
| --- | --- | --- | --- | --- | --- | --- | --- | --- | --- | --- |
| 1. Did the research question and inclusion criteria for the review include the components of PICO? |  | no | yes | yes | yes | yes | no | yes | yes | yes |
| 2. Did the report of the review contain an explicit statement that the review methods were established prior to the conduct of the review and did the report justify any significant deviations from the protocol? |  | no | yes | no | no | no | no | partial yes | yes | no |
|  |  |  |  |  |  |  |  |  |  |  |
| 3. Did the review authors explain their selection of the study designs for inclusion in the review? |  | no | yes | yes | yes | yes | no | yes | yes | no |
| 4. Did the review authors use a comprehensive literature search strategy? |  | partial yes | yes | partial yes | partial yes | partial yes | no | partial yes | yes | yes |
|  |  |  |  |  |  |  |  |  |  |  |
| 5. Did the review authors perform study selection in duplicate? |  | no | yes | yes | no | no | no | yes | yes | no |
| 6. Did the review authors perform data extraction in duplicate? |  | no | yes | yes | no | no | no | yes | no | no |
| 7. Did the review authors provide a list of excluded studies and justify the exclusions? |  | no | yes | yes | yes | no | no | yes | yes | partial yes |
|  |  |  |  |  |  |  |  |  |  |  |
| 8. Did the review authors describe the included studies in adequate detail? |  | no | yes | no | no | no | no | no | yes | yes |
|  |  |  |  |  |  |  |  |  |  |  |
| 9. Did the review authors use a satisfactory technique for assessing the risk of bias (RoB) in individual studies that were included in the review? | **RCT's** | no | partial yes | no |  | no |  |  | yes | yes |
|  | **NSRI** |  | partial yes |  | no |  | no | no | yes | Includes only RCTs |
| 10. Did the review authors report on the sources of funding for the studies included in the review? |  |  |  |  |  |  |  |  |  |  |
| 11. If meta-analysis was performed did the review authors use appropriate methods for statistical combination of results? | **RCT's** | No meta-analysis conducted | No meta-analysis conducted | No meta-analysis conducted | No meta-analysis conducted | No meta-analysis conducted | No meta-analysis conducted | No meta-analysis conducted | No meta-analysis conducted | No meta-analysis conducted |
|  | **NRSI** | No meta-analysis conducted | No meta-analysis conducted | No meta-analysis conducted | No meta-analysis conducted | No meta-analysis conducted | No meta-analysis conducted | No meta-analysis conducted | No meta-analysis conducted | No meta-analysis conducted |
| 12. If meta-analysis was performed, did the review authors assess the potential impact of RoB in individual studies on the results of the meta-analysis or other evidence synthesis? |  | No meta-analysis conducted | No meta-analysis conducted | No meta-analysis conducted | No meta-analysis conducted | No meta-analysis conducted | No meta-analysis conducted | No meta-analysis conducted | No meta-analysis conducted | No meta-analysis conducted |
| 13. Did the review authors account for RoB in individual studies when interpreting/ discussing the results of the review? |  | NA | yes | NA | NA | NA | NA | NA | yes | yes |
| 14. Did the review authors provide a satisfactory explanation for, and discussion of, any heterogeneity observed in the results of the review? |  | no | yes | yes | yes | yes | yes | yes | yes | yes |
| 15. If they performed quantitative synthesis did the review authors carry out an adequate investigation of publication bias (small study bias) and discuss its likely impact on the results of the review? |  | No meta-analysis conducted | No meta-analysis conducted | No meta-analysis conducted | No meta-analysis conducted | No meta-analysis conducted | No meta-analysis conducted | No meta-analysis conducted | yes | No meta-analysis conducted |
| 16. Did the review authors report any potential sources of conflict of interest, including any funding they received for conducting the review? |  | yes | no | yes | yes | no | no | no | yes | no |

Appendix 2 (continued)

| **Amstar Item** |  | **Rutten Abu-Omar, 2003** | **Stead, 2018** | **Thomas, 2018** | **WHO, 2009** |
| --- | --- | --- | --- | --- | --- |
| 1. Did the research question and inclusion criteria for the review include the components of PICO? |  | yes | yes | yes | yes |
| 2. Did the report of the review contain an explicit statement that the review methods were established prior to the conduct of the review and did the report justify any significant deviations from the protocol? |  | no | yes | no | yes |
| 3. Did the review authors explain their selection of the study designs for inclusion in the review? |  | no | yes | no | yes |
| 4. Did the review authors use a comprehensive literature search strategy? |  | no | yes | partial yes | yes |
| 5. Did the review authors perform study selection in duplicate? |  | no | yes | yes | no |
| 6. Did the review authors perform data extraction in duplicate? |  | no | yes | no | no |
| 7. Did the review authors provide a list of excluded studies and justify the exclusions? |  | no | yes | yes | partial yes |
| 8. Did the review authors describe the included studies in adequate detail? |  | no | yes | yes | partial yes |
| 9. Did the review authors use a satisfactory technique for assessing the risk of bias (RoB) in individual studies that were included in the review? | **RCT's** | yes | Includes only NRSI | no | yes |
|  | **NSRI** |  | yes |  | yes |
| 10. Did the review authors report on the sources of funding for the studies included in the review? |  |  |  |  |  |
| 11. If meta-analysis was performed did the review authors use appropriate methods for statistical combination of results? | **RCT's** | No meta-analysis conducted | No meta-analysis conducted | No meta-analysis conducted | No meta-analysis conducted |
|  | **NRSI** | No meta-analysis conducted | No meta-analysis conducted | No meta-analysis conducted | No meta-analysis conducted |
| 12. If meta-analysis was performed, did the review authors assess the potential impact of RoB in individual studies on the results of the meta-analysis or other evidence synthesis? |  | No meta-analysis conducted | No meta-analysis conducted | No meta-analysis conducted | No meta-analysis conducted |
| 13. Did the review authors account for RoB in individual studies when interpreting/ discussing the results of the review? |  | yes | yes | no | yes |
| 14. Did the review authors provide a satisfactory explanation for, and discussion of, any heterogeneity observed in the results of the review? |  | no | yes | NA | yes |
| 15. If they performed quantitative synthesis did the review authors carry out an adequate investigation of publication bias (small study bias) and discuss its likely impact on the results of the review? |  | No meta-analysis conducted | No meta-analysis conducted | No meta-analysis conducted | No meta-analysis conducted |
| 16. Did the review authors report any potential sources of conflict of interest, including any funding they received for conducting the review? |  | no | yes | yes | yes |

## Appendix 3: Typology of mass media campaigns to promote physical activity

| ***Policy level*** | **National** | 2 | 4 | 6 |
| --- | --- | --- | --- | --- |
|  | **Subnational (**local and regional, specify if possible**)** | 1 | 3 | 5 |
|  |  | **Awareness (indirect)** | **Behavioral intermediates (indirect)** | **Physical activity (direct)** |
|  |  | ***Level of impact*** | | |

## Appendix 4: Campaign characteristics

| Campaign | Included in n reviews | Typology campaign | Policy level | Country | Target population | Combined action /setting | Campaign year | Distal outcome (and which) | Intermediate outcomes | Proximal outcome | Effectiveness (+ / 0 / -) | Funder |
| --- | --- | --- | --- | --- | --- | --- | --- | --- | --- | --- | --- | --- |
| 10,000 steps Ghent | 4 [2-5] | 5 | Provincial / local | Belgium | Adults | Pedometer sale/loan, organization of community  2) Channel: local media campaign (not specified) events, community street signs | 2007-2010 OR 2005-2006/ (cocker vs Acker) | PA (pedometer & self-report) | NR | Awareness | + (PA)  + (Awareness) | Funded by the local government |
| 10,000 steps Rockhampton | 3 [3, 5, 6] | 5 | Local | Australia | Adults | Community-based activities, environmental prompts, e-mails and newsletters | 2001-2003 | PA (self-report) | NR | Awareness | 0 (PA)  + (awareness) | Funded by the local government |
| 5-4-3-2-1 Go! | 1 [2] | 5 | Local / municipal | US | Children | Brochure, bottle, magnet | NA | PA (self-report) | Assessed but not specified | NR | 0 (PA)  +/- (intermediate) | Consortium to Lower Obesity in Chicago Children (CLOCC),  Chicago department of public health,  Chicago department of youth services,  Chicago Community in Schools (CCIS),  PepsiCo Foundation |
| Activate Omaha | 1 [5] | 3 & 5 | Local | US | Adults | Social marketing, bilboards, newspaper adverts, commercial, website | 2005 (8 weeks) -2007 (12 weeks) | PA (explore Omaha on foot) | Knowledge, belief | NR | + (PA)  + (intermediate) | Federal government, as well as private funding (American heart association, Amy L. Scott Foundation, Area Health Education Consortium, Blue Cross Blue Shield NE, Nebraska Health & Human Services, Omaha Community Foundation, Papio Natural Resource District, Sarpy Cass County Health & Wellness Department, Union Pacific Railroad, Wild Oats) |
| Active Australia | 3 [6-8]  8 [3, 6, 9-14] | 5 | Local (statewide) | New Zealand & Australia | Adults | NA | 1997-1999 | PA | Knowledge, attitude | Awareness, recall | + (PA)  + (intermediate)  + (proximal) | NR |
| Active for Life | 7 [6, 7, 9, 10, 12, 13, 15] | 6 | National | UK | Adults | Linked to professional education programme | 1996-1998 | PA, sedentary behavior | Knowledge | Awareness, recall | 0 (PA)  + (knowledge)  + (awareness) | Department of health |
| Agita Sao Paolo | 4 [5, 6, 14, 16] | 1 & 5 | Local | Brazil | Students, workers, elderly | Community events, free media coverage, posters, flyers, brochures | 1996-2008 | PA (self-report?) | NR | Awareness, recall | + (PA)  + (proximal) | State secretariat of Health, partner institutions, and private funding |
| BC Walks | 4 [5, 7, 14, 17] | 1 & 5 | Local | US | Insufficiently active 40-65 year olds | PA logbook (website to log PA), Commercials, radio, advertisement, website, additional print for African Americans | 2003 (8 weeks) | PA (walking, mvpa) | Knowledge | Awareness | + (PA)  + (awareness) | CDC |
| Wheeling Walks | 10 [2, 3, 5-7, 11, 12, 14, 17, 18] | 5 | Local (municipal) | US | Sedentary adults (50-65 year olds) | Community campaign, including educational  activities at  worksites,  churches and  other organizations, website,  PA Taskforce,  environmental  changes | 2001-2002, 2011-2012 | PA (self-report walking) | Intention | Awareness, recall | + (PA)  + (intention)  + (Awareness) | Private foundation (Robert Wood Johnson Foundation),  Community foundation (the Claude Worthington Benedum Foundation),  Corporate foundations (WesBanco, Wheeling Hospital, and Ohio Valley Health Services and Education Corporation),  Local, state, federal government (Wheeling Ohio-County Health Department, the West Virginia Bureau for Public Health) |
| West Virginia Walks | 3 [5, 7, 17] |  | Local | US | 40-65 yr old | Commercial, radio, website | 2005 (8 weeks) | PA (walking, MVPA) | NR | Awareness | + (PA)  + (proximal) | NR |
| Welch walks | 1 [4] | 5 | Local | US | Adults | NR | 2002, 2-week campaign | PA (walking) | NR | NR | 0 | NR |
| Canada on the move | 6 [5, 7, 8, 10, 14, 17] | 6 | National | Canada | General population, >18 yr | PA logbook (website for logging steps), free mass distribution of pedometers in cereal box | 2003-2004 | PA (walking) | Intention | Awareness, recall | + (PA)  + (awareness) | NR |
| Yuma on the move | 4 [6, 10-12] | 5 | Local | US | Adults, in  precontemplation/ contemplation  stage of change | Linked to  local competitions,  worksite  promotions | 1997-1999, 2yr | PA | Knowledge, intention, self-efficacy | Recall | + (PA)  + (intermediate)  + (recall) | CDC |
| Getting Switzerland on the Move – Sports for All | 1 [6] | 6 | National | Switzerland | Inactive people | Training and information courses in sports clubs | 3 yrs duration | PA | NR | NR | + (PA) | Swiss health insurance companies together with the Swiss Olympic Association |
| Finnish experience: Finland on the Move & Fit for Life | 1 [6] | 6 | National / regional | Finland | Finland on the move: General population,  Fit for Life: 40-60 yr old | Both: Support on the local and regional level (financial, training and consultation) | Finland om the move: 1991-1994 (4yr),  Fit for life: 5yr | PA (leisure, diary, | NR | NR | + (PA) | Fit for life: Ministry of education and Ministry of social affairs and health, annual buget: 770 000 US$ |
| Change 4 Life | 3 [2, 8, 19] | 6 | National | UK | Parents of 5-11 year olds, low SES | Helpline, website, sponsors | 2009-2011 | PA | Knowledge, attitude, intention | Awareness | 0 (PA)  +/- (intermediate)  + (proximal) | Engagement with  partners and  workforces, local  service providers,  potential local  supporters, and  NGOs |
| Concord, a Great Place to be Active | [6] | 5 | Local | Australia | Women 20-50 years old | Walking groups, community events, print materials | 1997-1999, 2yrs | SB | Attitude | NR | + (SB)  + (attitude) | NSW Health Department, as part of the Physical Activity Demonstration Grant Scheme |
| Exercise: make it part of your day | 5 [8, 10, 11, 20, 21] | 6 | National | Australia | Adults >15 yr | Links to community activities such  as publicity and  events, physical  activity days,  competitions  during ‘Heart  Week’ | 1 week in 1990 | PA (walking) | Knowledge, beliefs, attitude | Recall | +/- (PA only in lowest educated)  0 (beliefs)  + (recall) | NR |
| Exercise: take another step (builds on ‘Exercise: make it part of you day’) | 3 [8, 11, 20, 21] | 6 | National | Australia | Adults >15 yr | Community activities, physician education, ‘Heart week’ campaign | NR | PA (walking), SB | Intention | Recall | 0 (PA, SB)  0 (intention)  + (recall) | NR |
| Fighting fat, fighting fit | 6 [6, 8, 10-12, 19] | 6 | National | UK | Adults, targeted to overweight/obese, low SES | Little community activity, offered incentives to use local fitness centres | 1999 | PA | NA | Awareness | + (PA)  + (Awareness) | BBC |
| Fighting obesity campaign | 1 [19] | 6 | National | Turkey | Adults | TV, Print,  Outdoor, Radio, Website | 2010-2014 | PA | Knowledge | Awareness | +/- (PA)  + (knowledge)  + (Awareness) | NR |
| Find thirty every day / finding thirty it’s not a big exercise | 2 [2, 6, 8] | 5 | Provincial | Australia | Adults, low SES | TV, radio, print, billboard | 2008-2009 | PA | Knowledge, attitude etc | Awareness | + (PA)  + (knowledge)  + (Awareness) | NR |
| Get Fit with the Corren | 2 [11, 21] | 5 | Local | Sweden | Adults, readers of the Corren newspaper | Fitness test offered to participants | 1977-1978 (12 months) | PA | NR | Awareness | + (PA among registered)  + (awareness) | NR |
| Get up and do something | 3 [5, 10, 17] | 1 & 3 | Local | US | 18-30 yr old | Commercials, bus wraps, billboards, radio, website | 2001 (13 weeks) | NR | Intention | Recall | + (intermediate)  + (distal) | NR |
| Healthy children Healthy futures | 1 [14] | 3 | Local (3 cities) | US | Underrepresented minority youth | Target messaging at schools, parent organizations, community events, conferences, and child health clinic waiting areas | 2005 (6 month) | NR | Knowledge | NR | + | MetLife Foundation |
| Healthy People 2000 (HP2000) | 1 [6] | 4 | National | US | New exercisers, or who desires to adopt regular PA | Partnership hospital, community parks and recreation department, annual event | 2000 (8 weeks) | NR | Self-efficacy | NR | + (in some stages of change) | CDC, Department of Health and Human Services |
| Healthy U | 1 [5] | 1 | Regional | Canada | Adults 55-70 yr old | Commercials, website | 2007 (8 weeks) | NR | NR | Awareness | + (awareness) | Publicly funded |
| Heart to Heart project | 1 [14] | 6 | Regional | US | NR | Community activity | 1995 (4 month campaign, for 5 yrs) | PA (inactivity) | Knowledge | Awareness (of cvd) | 0 (PA)  + (intermediate, proximal) | NR |
| Heartbeat Wales | 3 [6, 14, 21] | 6 | Local | Wales, UK | General population | Educational promotions, e.g. at workplace | 1985 and 1990 | PA | Assessed but not specified | Assessed but not specified | 0 (PA)  0(intermediate)  0 (proximal) | Welsh Office |
| HEBS Walking campaign | 6 [6, 8, 11, 13, 20, 21] | 6 | National | Scotland | Adults (30-55yr), low SES | Little community activity | 1995-1996 | Walking | Intention, knowledge | recall | 0 (PA)  + (intermediate)  + (proximal) | Health Education Board for Scotland |
| Illawarra physical activity project | 1 [6] | NR | Local | Australia | NR | NR | NR | NR | NR | NR | NR | NR |
| Educational Interventions to Promote Healthy Nutrition and Physical Activity Among Older Chinese Americans | 1 [22] | 5 | Local | US | Older Chinese Americans | Lectures | 2010-2013 | PA, adherence to recommendations | Knowledge | NR | + (PA)  + (intermediate) | NR |
| Isfahan Healthy Heart Program | 3 [6, 14, 21] | NA | National | Iran | specific target groups like school  children, women, work-site, health personnel, high-risk persons | Target at individual, group  and community levels, using  mass media, health services,  community partnerships,  policies and legislation | 1999-20005 | NR | NR | NR | NA | management and planning department of the  National Organization of Budget and Management, Deputy  of Health in the Ministry of Health, Cardiovascular Research  Center, Isfahan Provincial Health Center and Isfahan University of Medical Sciences |
| otsego-schoharie healthy heart program | 2 [6, 14] | 5 | Local | US | Rural counties | Radio, local walking groups promoted, fact sheets at workplaces, school, supermarkets, medical practices | 1989-1994 | Sedentary behavior | NR | NR | + (PA) | Federal funding, New York state department of health, mary lasker heart and hypertension institute |
| Large scale, short-term, media-based weight loss program | 2 [10, 21] | NR | Local | US | Obese adults | Encouragement to attend self-help groups dealing with obesity | 1986 (3 weeks) | NR | NR | NR | NR | NR |
| Let’s GO! 5-2-1-0 | 1 [2] | 5 | Local | US | Children | Collaboration with school based programs, worksites, environmental and policy support | 2007-2011 | PA | Knowledge, intention | Awareness/ recall | +/- (PA)  +/- (intermediate)  + (proximal) | American Academy of Pediatrics; the Anthem Foundation; the United States Department of Health and Human Services, Centers for Disease Control and Prevention, private health foundations |
| LiveLighter | 1 [19] | 6 | Local | Australia | Adults (25-64 yr) | Advocacy to generate support for policy and environmental changes | 2012 (3 month) | PA | Intention | Recall | 0 (PA)  + (intermediate)  + (proximal) | Government of Western Australia, Department of Health, cancer council |
| Maak je niet dik | 3 [2, 8, 19] | 2 | National | The Netherlands | General population | NA | 2002-2004, 2007-2010 | PA | attitude | Awareness/ recall | 0 (PA)  + (intermediate)  + (proximal) | The Netherlands Nutrition Centre, government funded organization |
| Measure-Up | 2 [2, 8] | 6 | National | Australia | General population | Local activities to support campaign, distribute campaign materials, support public relations in local events | 2008-2009 | PA | Knowledge, attitude | Recall | + (PA, by awareness)  + (intermediate)  + (proximal) | Government |
| Bootheel Heart Health Project | 1 [4] | 5 | Local | US | Adults (>18 yr) | Tailored newsletters, interpersonal activities on social support, community wide events (walk-a-thons) | 1989 (3 yr) | Walking | NR | NR | + (PA) | Centers for Disease Control and Prevention (CDC) and the Missouri Department of  Health |
| Minnesota Heart Health Program | 3 [11, 20, 21] | 5 | Local | US | Adults (24-74 yr) | Strong community components, health professional education, screening, counseling | 1987 -1994 (annual 1-3 month campaign) | PA (self-report) | NR | Awareness | + (PA)  + (proximal) | National Heart,  Lung and Blood, National Institutes of Health |
| Slagerup – a heart healthy town | 3 [11, 20, 21] | 5 | Local | Denmark | Adults (20-6 yr) | Link to community; fitness tests, lectures, heart day | 1989 | PA | Intention | Awareness | 0 (PA)  + (intermediate) | NR |
| New South Wales Walk Safely to School Day | 1 [10] | 5 | Local | Australia | Adults | Parent and school programs | 2001-2004 (annually 3 week) | PA | NR | NR | 0 (PA) | NSW health |
| Pawtucket Heart Health Program | 1 [6] | 5 | Local | US | Adults | NR | 1983-1991 | PA | NR | NR | 0 (PA) | National Heart, Lung, and Blood Institute |
| Substudy within the Pawtucket Heart Health Program | 1 [20] | 3 | Local | US | NR | NR | (6 weeks) | NR | Motivation | NR | + (intermediate) | NR |
| Muévete Botoá | 1 [5] | 5 | Local | Columbia | Adults >18 yr old | Programs to change PA in variety of settings, partnerships with professionals, education, community members | 1998 – ongoing in 2011 | PA | NR | NR | 0 (PA) | Private and public |
| My Participaction | 1 [2] | 6 | National | Canada | Adults | NR | 2010 (33 weeks) | PA | Knowledge, attitude | Awareness/ recall | + (PA by intermediate)  + (intermediate by awareness)  + (proximal) | NR |
| ParticipACTION | 2 [5, 8] | 6 | National | Canada | Parents of 7-12 yr olds | English and French messages, commercials | 2007-2008 (6 months) | PA | Knowledge | Awareness | + (PA)  + (intermediate)  + (proximal) | Public and private funding |
| Think Again, Participaction | 1 [2] | 6 | National | Canada | Children (parents) | NR | 2011-2012 | PA | Knowledge, attitude | Awareness / recall | +/- (PA)  + (intermediate by awereness)  0 (proximal) | NR |
| Heartfile | [14] | 6 | National | Pakistan | Newspaper readers | Outreach at local level | 1999-2001 | PA | Knowledge | NR | + (PA)  + (intermediate) | NR |
| Netherlands on the Move | 1 [7] | 6 | National | The Netherlands | Main focus older adults (>50), but >35 yr included | Broadcasted exercise and health education program | 2000 | SB | Knowledge | NR | + (SB)  + (intermediate) | NOC*NSF (Netherlands Olympic Committee, Netherlands Sport federations) |
| Physical activity: the arthritis pain reliever | [2] | 5 | Local | US | Adults | Fitness fair, walk and talk (public relations event), lectures for older adults | 2003 (spring) | PA | Knowledge, attitude | Awareness / recall | + (PA)  0 (intermediate)  0 (proximal) | CDC |
| Push Play | 5 [5, 7, 10, 14, 17] | 6 | National | New Zealand | Adults, males 30-54 yr old | National Push Play day, commercials, billboards, radio, magazine | 1999-2001 | Sedentary behavior | Intention | Awareness | + (SB short-term)  0 (SB, long term)  + (intermediate)  + (Awareness) | Hillary Commision (public funding agency) |
| Project WOW (Walk the Ozarks to Wellness) | 1 [4] | 5 | Local | US | Adults | Tailored newsletters, interpersonal activities, health counsling | 2003-2004 | Walking, PA | NR | NR | + (PA) | CDC, National institutes of health |
| Romsas in motion | 1 [7] | 5 | Local | Norway | Adults (30-67 yr) | Communication, physical activity programs, environmental strategies, participatory strategies | 2000-2003 | SB | NR | NR | + (SB) | Norwegian Institute of Public  Health, the Directorate for Health and Social Affairs, the Norwegian  Research Council, the Norwegian Foundation for Health and Rehabilitation, and the Romsås District Administation |
| Sogn og Fjordane County campaign | 1 [11] |  | Local | Norway | Adults (16-68 yr) | Community action | ±1983 (1 week) | Tried new activity | NR | Awareness | + (PA)  + (Awareness) | NR |
| Stanford 5 City community-wide cvd risk reduction project | 4 [11, 18, 20, 21] | 5 | Local | US | General population (12-74 yr) | Community based components: talks, seminars, workshops, walking groups, worksite programs, competitions | 1980 (6 yr) | PA | Knowledge, attitudes, self-efficacy | NR | + (PA)  0 (intermediate) | US Public Health Service, the National Heart, Blood  and Lung Institute |
| Stanford Heart Disease Prevention Program | 3 [10, 11, 21] | 5 | Local | US | Adults (35-59 yr) | Bilingual campaign | 1980 (2 yr) | PA | NR | NR | 0 (PA) | Stanford Heart  Disease Prevention Program, the Lipid Research Clinic at Stanford University |
| Stay Active Stay independent | 3 [5, 10, 17] | 5 | Local | Australia | Older adults, 60-75 yr old | Community partnerships | 2004-2005 (18 month) | PA | Intention, interest | Awareness | + (PA)  + (intention)  + (awareness) | NR |
| Step it up Hawaii / Start living healthy/ Healthy Hawaii initiative | 3 [2, 5, 8] | 3 | local | US | Adults 35-55 yr, low SES | Website, supermarket partnership, health fairs and community events, school walking campagin | 2007 (10 weeks) | NR | Knowledge, attitude | Awareness, recall | + (intermediate)  + (proximal) | Hawaii State Department of Health, Healthy Hawaii Initiative |
| Step Up. Step Out | 2 [2, 3] | 5 | Local | US | Women | Pedometer, goal setting guides, newsletters, groups events, incentive prizes | 2004-2005 | PA | Knowledge, attitude | NR | + (PA)  +/- (intermediate) | Community-based participatory research (CBPR), e Centers for Disease Control and Prevention  Office of Science and Extramural Research |
| Steps to a Healthier New Orleans /"Treat you Right" | [5] |  |  | US | African American women, 18-49 yr old | Commercials, radio, bus signs | 2005 (6 months) | PA (leisure and utilitarian walking) | Attitude | Recall | 0 (PA)  + (intermediate)  + (proximal) | NR |
| COMMUNICATE | 1 [2] | 5 | Local | Japan | Older adults (40-79 yr) | Health education program, support delivery | 2009-2012 | PA | Knowledge, attitude | Awareness, recall | + (PA)  +/- (intermediate)  + (proximal) | Ministry of Health, Labour and Welfare of Japan |
| The Gangwon Province Health Day Walking Campaign | 1 [2] |  | Local | Korea | General population | NA | 2007 (2 weeks) | PA | Knowledge, attitude | NA | + (PA)  +/- (intermediate) | Provincial government |
| The Long Live Kids | [2] | 6 | National | Canada | Children | NA | 2004-2007 | PA | Intention | Awareness, recall | +/- (PA)  0 (intermediate)  + (proximal) | NR |
| Sister talk | 1 [22] | 5 | Local | US | African American women | Toll-free number to call, support calls | ±2013 (12 weeks) | PA (leisure) | NR | NR | + (PA) | National Cancer Institute |
| Tu Salud, ¡Si Cuenta! (Your health matters) | 1 [2] | 5 | Local | US | Adults | Newsletter, community health worker outreach, free exercise classes, walking trail, weight loss challenge | 2005-2010 | PA | NA | NR | + (PA by awareness) | UT Health Clinical and Translational Science Award, NIH/National Center on Minority Health and Health Disparities, and the Texas Department of State Health Services funding for University of Texas Community Outreach |
| VERB | 5 [2, 10, 14, 16, 18] | 6 | National | US | Tweens (9-12yr) | Supporting in-school promotion, radio and internet. | 2003 (1 yr) | PA | NR | Awareness | + (PA)  + (awareness) | CDC |
| Walk Missouri | [5] | 5 | Local | US | General population | Radio, billboards, newspaper adverts, posters | 2003 (5 months) | PA (walking days) | Beliefs | Recall | + (PA)  + (intermediate)  + (recall) | NR |
| Walk to Work | [5] | 6 | National | Australia | Urban dwelling adults, 18-65 yr old | Newspaper advert, commercials, radio, PR, government intranet network | 2003 (8 week) | PA (active commute) | NR | Recall | + (PA)  + (recall) | NR |
| Weight of the Nation | 1 [19] | 3 | Local | US | General population | Community based outreach campaign, local screening events | 2012 | NR | Self-efficacy, intention | NR | + (intermediate) | HBO, CDC, Institute of Medicine, NIH, Michael & Susan Dell Foundation, and Kaiser Permanente |

Outcome effect categories: +/- indicate mixed results, 0 indicates no effect, + indicates a beneficial effect

# References

1. Knowler, W.C., et al., *Reduction in the incidence of type 2 diabetes with lifestyle intervention or metformin.* N Engl J Med, 2002. **346**(6): p. 393-403.

2. Yun, L., et al., *A Systematic Review of Community-wide Media Physical Activity Campaigns: An Update From 2010.* J Phys Act Health, 2017. **14**(7): p. 552-570.

3. Anker, A.E., et al., *Measuring the Effectiveness of Mass-Mediated Health Campaigns Through Meta-Analysis.* Journal of Health Communication, 2016. **21**(4): p. 439-456.

4. Foster, C., et al., *What works to promote walking at the population level? A systematic review.* Br J Sports Med, 2018. **52**(12): p. 807-812.

5. Leavy, J.E., et al., *Physical activity mass media campaigns and their evaluation: a systematic review of the literature 2003-2010.* Health Education Research, 2011. **26**(6): p. 1060-1085.

6. Marshall, A.L., N. Owen, and A.E. Bauman, *Mediated approaches for influencing physical activity: update of the evidence on mass media, print, telephone and website delivery of interventions.* J Sci Med Sport, 2004. **7**(1): p. 74-80.

7. Abioye, A.I., K. Hajifathalian, and G. Danaei, *Do mass media campaigns improve physical activity? a systematic review and meta-analysis.* Arch Public Health, 2013. **71**(1): p. 20.

8. Thomas, M.M., et al., *A review of the impact of physical activity mass media campaigns on low compared to high socioeconomic groups.* Health Educ Res, 2018. **33**(5): p. 429-446.

9. Abu-Omar, K. and A. Rütten, *[Physical activity and public health].* Bundesgesundheitsblatt Gesundheitsforschung Gesundheitsschutz, 2012. **55**(1): p. 66-72.

10. Brown, D.R., et al., *Stand-alone mass media campaigns to increase physical activity: a Community Guide updated review.* Am J Prev Med, 2012. **43**(5): p. 551-61.

11. Cavill, N. and A. Bauman, *Changing the way people think about health-enhancing physical activity: do mass media campaigns have a role?* J Sports Sci, 2004. **22**(8): p. 771-90.

12. Finlay, S.J. and G. Faulkner, *Physical activity promotion through the mass media: inception, production, transmission and consumption.* Prev Med, 2005. **40**(2): p. 121-30.

13. Rütten, A. and K. Abu-Omar, *The evidence-base for interventions to promote physical activity.* Zeitschrift fur Gesundheitswissenschaften, 2003. **11**(3): p. 229-246.

14. *WHO Guidelines Approved by the Guidelines Review Committee*, in *Interventions on Diet and Physical Activity: What Works: Summary Report*. 2009, World Health Organization Copyright © World Health Organization 2009.: Geneva.

15. Cavill, N., *National campaigns to promote physical activity: can they make a difference?* Int J Obes Relat Metab Disord, 1998. **22**: p. S48-51.

16. Pate, R.R., et al., *Policies to Increase Physical Activity in Children and Youth.* Journal of Exercise Science & Fitness, 2011. **9**(1): p. 1-14.

17. Bauman, A. and J. Chau, *The Role of Media in Promoting Physical Activity.* J Phys Act Health, 2009. **6**: p. S196-s210.

18. Heath, G.W., *The role of the public health sector in promoting physical activity: national, state, and local applications.* J Phys Act Health, 2009. **6**: p. S159-67.

19. Kite, J., et al., *A Systematic Search and Review of Adult-Targeted Overweight and Obesity Prevention Mass Media Campaigns and Their Evaluation: 2000-2017.* Journal of Health Communication, 2018. **23**(2): p. 207-232.

20. Marcus, B.H., et al., *Physical activity interventions using mass media, print media, and information technology.* American Journal of Preventive Medicine, 1998. **15**(4): p. 362-378.

21. Kahn, E.B., et al., *The effectiveness of interventions to increase physical activity. A systematic review.* Am J Prev Med, 2002. **22**(4): p. 73-107.

22. Mosdøl, A., et al., *Targeted mass media interventions promoting healthy behaviours to reduce risk of non-communicable diseases in adult, ethnic minorities.* Cochrane Database Syst Rev, 2017. **2**(2): p. Cd011683.
